# Characterization of *Polaribacter formosus* sp. nov. and *Polaribacter salmoneus* sp. nov. with Comparative Genomic Analysis of the Genus *Polaribacter*

**DOI:** 10.3390/microorganisms14102227

**Published:** 2026-10-02

**Authors:** Dandan Zhang, Yuqi Ye, Siyu An, Fang Luan, Zongjun Du

**Affiliations:** 1 Naval Architecture and Port Engineering College, Shandong Jiaotong University, Weihai 264209, China; 220161@sdjtu.edu.cn (D.Z.); 221010@sdjtu.edu.cn (F.L.); 2 Marine College, Shandong University, Weihai 264209, China; yeyuqi2023@163.com; 3 Jilin Province Product Quality Supervision and Inspection Institute, Changchun 130103, China; ansiyu1991@163.com; 4 Shandong Key Laboratory of Intelligent Marine Engineering Geology, Environment and Equipment, Qingdao 266237, China

**Keywords:** *Polaribacter formosus* sp. nov., *Polaribacter salmoneus* sp. nov., polyphasic taxonomy, cadmium-resistance analysis, comparative genomic analysis

## Abstract

Two novel Gram-negative, rod-shaped, carotenoid-pigmented strains, Z022^T^ and Z014^T^, were isolated from the viscera of abalone, *Haliotis discus hannai*, in Weihai, China and from coastal sediment samples collected at the Antarctica Great Wall station, respectively. Phylogenetic analysis of the 16S rRNA gene sequences and the 120 ubiquitous single-copy proteins showed both the strains belonged to the genus *Polaribacter*. Genomic analysis, including the average nucleotide identity (ANI), digital DNA-DNA hybridization (dDDH), amino acid identity (AAI), and percentage of conserved proteins (POCP) among Z022^T^, Z014^T^ and the reference strains suggested that the strains were two novel species within the genus *Polaribacter*. Pan-genome analysis revealed that 1079 core genes were shared among the 33 *Polaribacter* genomes. Biogeographic distribution analysis indicated that the marine environments are the primary habitat of the *Polaribacter* strains. The strain Z014^T^ could tolerate up to 0.3 mM CdCl_2_ under the tested conditions. The predominant cellular fatty acids of strain Z022^T^ were iso-C_15:0_ and iso-C_15:0_ 3-OH; the predominant cellular fatty acids of strain Z014^T^ were iso-C_15:0_ 3-OH, iso-C_15:0_ and C_15:0_ 3-OH. The major polar lipids of strain Z022^T^ were phosphatidylethanolamine (PE), aminolipid (AL) and three unidentified lipids (L1-3). The major polar lipids of strain Z014^T^ were aminolipid (AL), aminoglycolipid (AGL) and four unidentified lipids (L1-4). According to the phenotypic, chemotaxonomic characterization, phylogenetic classification and genome analysis, Z022^T^ and Z014^T^ are identified as two novel species of the genus *Polaribacter*, for which the names *Polaribacter formosus* sp. nov. and *Polaribacter salmoneus* sp. nov. are proposed with the type strain Z022^T^ (=MCCC 1H00505^T^ = KCTC 92009^T^) and Z014^T^ (=MCCC 1H00504^T^ = KCTC 92010^T^), respectively.

## 1. Introduction

The genus *Polaribacter*, belonging to the family *Flavobacteriaceae*, was first proposed by Gosink et al. in 1998 [1], with the description of *Polaribacter filamentus* (type species), *Polaribacter franzmannii* and *Polaribacter irgensii*. At the time of writing, there are 34 species with validly published names, according to the List of Prokaryotic Names with Standing in Nomenclature (LPSN, https://lpsn.dsmz.de/genus/polaribacter (accessed on 1 May 2026)), belonging to the genus *Polaribacter* [2]. Members of the genus *Polaribacter* are Gram-stain-negative and aerobic. Most species are non-motile and rod-shaped, and can form yellow-to-orange-pigmented colonies [3,4]. Menaquinone-6 (MK-6) was the major respiratory quinone of the genus *Polaribacter*. Species were all isolated from the marine-related environments, including the polar regions [1,5], marine animals [6,7], algae [8,9] and sea water [10,11].

Members of the genus *Polaribacter* are recognized as important degraders of high-molecular-weight organic matter in marine environments, particularly complex polysaccharides released during algal blooms, and they often become dominant taxa under such conditions [12]. These ecological features suggest that *Polaribacter* strains possess diverse adaptive strategies to marine environments. In this paper, two novel strains—Z022^T^, isolated from viscera of abalone, and Z014^T^, isolated from the sediment samples collected from the Antarctica Great Wall station—are characterized. Based on the phenotypic, chemotaxonomic and phylogenetic analyses of Z022^T^ and Z014^T^, *Polaribacter formosus* sp. nov. and *Polaribacter salmoneus* sp. nov. are proposed.

## 2. Materials and Methods

### 2.1. Bacterial Isolation and Cultivation

Strain Z022^T^ was isolated from the viscera of abalone, *Haliotis discus hannai*, in Rongcheng, Shandong Province, China (122°14′ E, 36°54′ N). The abalone was dissected by a sterile scalpel to expose the viscera; then the viscera were thoroughly ground to obtain the visceral sample. One gram of the visceral sample was added to 100 mL sterile seawater, and the mixture was incubated at 20 °C and 150 rpm. The sample was serially diluted to 10^−4^ with sterile seawater, and 0.1 mL aliquots of each dilution were spread onto marine agar 2216 (MA; Becton Dickinson, Franklin Lakes, NJ, USA). After the incubation at 30 °C for 5 days, one light yellow colony was obtained and designated as Z022^T^. Strain Z014^T^ was isolated from coastal sediment samples collected at the water depth of 95 m near the Antarctica Great Wall station (58°55′ W, 62°13′ S) in February 2018. A quantity of 1 g of the sediment sample was added into 100 mL sterile seawater and the mixture was serially diluted to 10^−3^, and 0.1 mL aliquots of each dilution were spread onto MA. The plates were incubated at 15 °C for 15 days, and one salmon-colored colony was obtained and designated as Z014^T^. Both strains were stored at −80 °C in sterile 15.0% (*v*/*v*) glycerol supplemented with 3.0% (*v*/*v*) NaCl. The type strain *P. filamentus* KCTC 23135^T^ was obtained from the Korean Collection for Type Cultures (KCTC) and cultured at 4 °C on MA. The type strain *P. atrinae* JCM 19202^T^ was obtained from Japan Collection of Microorganisms (JCM), and cultured at 20 °C on MA.

### 2.2. The 16S rRNA Gene Sequencing and Phylogenetic Analysis

The 16S rRNA gene was amplified by polymerase chain reaction (PCR) using two universal primers: 27F and 1492R [13]. Then the clone operation was performed as previously described [14]. The 16S rRNA gene was sequenced by Ruibotech (Qingdao, China). Similar sequences of strains Z022^T^ and Z014^T^ were searched for on the EzBioCloud server (http://www.ezbiocloud.net/, accessed on 1 May 2026) and the NCBI database (https://blast.ncbi.nlm.nih.gov/Blast.cgi, accessed on 1 June 2026). Phylogenetic trees were constructed using the neighbor-joining (NJ) [15], the maximum-likelihood (ML) [16] and maximum-parsimony (MP) [17] methods implemented in MEGA X (version 10.2) [18]. Bootstrap values were determined based on 1000 replicates for each of the three methods. K2 + G + I was the best-fit substitution model to construct the ML tree.

### 2.3. Whole-Genome Sequencing, Genomic and Phylogenomic Analyses

The genomic DNA of strains Z022^T^ and Z014^T^ was extracted according to the manufacturer’s instructions of the Tiangen Bacterial DNA Kit and sequenced by Beijing Novogene Biotechnology Co., Ltd. (Beijing, China) on the NovaSeq 6000 platform (Illumina, San Diego, CA, USA). The raw reads were generated by the base-calling software CASAVA (version 1.8) and filtered using the data quality control software Fastp (version 0.23.0). High-quality clean data were assembled using the software SOAPdenovo2 (version 2.04), SPAdes (version 3.10.0) and ABySS (version 1.3.7). The assembly results from the three software packages were integrated using CISA (version 1.3), and the assembly with the fewest scaffolds was selected. GapCloser (version 1.12) was used to fill gaps, and reads with sequencing depth below 0.35× the average were removed to eliminate same-lane contamination. Genomes of strains belonging to the genus *Polaribacter* were downloaded from the NCBI prokaryotic reference genome database.

The gene function was annotated with the NCBI Prokaryotic Genome Annotation Pipeline [19,20] and the genes included in the metabolic pathways were analyzed with the KEGG database [21]. The ContEst16S was used to check the contamination of Z022^T^ and Z014^T^ genomes. The CheckM web server (https://nmdc.cn/analyze/classification (accessed on 21 September 2026)) was used to assess the completeness and contamination of the Z022^T^ and Z014^T^ genomes.

The average nucleotide identity (ANI) was calculated with a Web service (https://www.ezbiocloud.net/tools/ani (accessed on 15 June 2026)) [22] between strains Z022^T^, Z014^T^ and the type strains. The digital DNA-DNA hybridization (dDDH) was also calculated with the Genome-to-Genome Distance Calculator (GGDC) (http://ggdc.dsmz.de/ggdc.php/ (accessed on 16 June 2026)). The average amino acid identity (AAI) and the percentage of conserved proteins (POCP) were calculated with https://github.com/2015qyliang/POCP (accessed on 16 June 2026) with the version of v2.1. The concatenated alignment sequences of 120 ubiquitous single-copy proteins were obtained with GTDB-Tk (version 1.3.0) [23], and the phylogenetic tree was reconstructed with IQ-TREE [24] using the YEAST + F + I + G4 model with 1000 bootstrap replicates.

### 2.4. Comparative Genomic Analysis and Biogeographic Distribution of the Genus Polaribacter

The genome information used in this article within the genus *Polaribacter* is listed in Supplementary Table S1. The metabolic pathways were annotated with Rapid Annotation using Subsystem Technology (RAST) version 2.0 and the KEGG’s BlastKOALA server [25]. In order to evaluate the gene diversity and adaptive potential of the strains among the genus *Polaribacter*, the core and unique genes were analyzed with the Bacterial Pan Genome Analysis (BPGA) tool with the threshold of 50% amino acid sequence identity [26].

The global distribution and habitat preference of the genus *Polaribacter* were analyzed with the Microbe Atlas Project (MAP, https://microbeatlas.org/, accessed on 24 May 2026) with a 99% sequence similarity threshold.

### 2.5. Morphological, Physiological and Biochemical Analyses

Cells of strains Z022^T^ and Z014^T^ grown on MA at 28 °C and 25 °C for 3 days were used for morphological and physiological tests. Cell morphology and size were examined with a scanning electron microscope (Nova NanoSEM 450; FEI, Hillsboro, OR, USA). Gram staining of strains Z022^T^ and Z014^T^ was assessed as described by Smibert and Krieg [27], and the examination of gliding motility was carried out according to the hanging-drop method [28]. The temperature ranges for growth of strains Z022^T^ and Z014^T^ were evaluated at 0, 4, 15, 25, 28, 30, 33, 37, 40, 42 and 45 °C on MA, and results were recorded every 12 h. The effect of NaCl concentration on the growth of strains Z022^T^ and Z014^T^ was examined on MA, which was prepared with artificial seawater (0.23% MgCl_2_, 0.32% MgSO_4_, 0.12% CaCl_2_, 0.07% KCl, and 0.02% NaHCO_3_, all *w*/*v*) and supplemented with different concentrations of NaCl (0%, 0.5%, and 1–10% in 0.5% increments, *w*/*v*). The pH range for growth was determined using modified MB (30 g sea salt [Sigma, St. Louis, MO, USA] L^−1^, 1 g yeast extract L^−1^, 5 g peptone L^−1^, and 0.1 g ferric citrate L^−1^; pH 7.5) with different buffers [MES (pH 5.5 and 6.0), PIPES (pH 6.5 and 7.0), HEPES (pH 7.5 and 8.0), Tricine (pH 8.5) and CAPSO (pH 9.0, 9.5 and 10.0) (Sangon, Shanghai, China)] at pH 5.5–10.0 (in 0.5 unit intervals). The buffers were added to different levels at concentrations of 20 mM, and the pH of the medium was adjusted by adding 1 M HCl or NaOH before autoclaving. Then ranges of pH were investigated on 96-well microplates by measuring the OD_600_. A 1 M cadmium chloride stock solution was prepared with the deionized water and filtered with the 0.2 μm nitrocellulose filter. The Cd^2+^ resistance of the two strains was examined on the MA in the presence of 0.0–0.5 mM cadmium chloride at intervals of 0.1 mM. A 100 μL aliquot of the cell suspension (0.5 McFarland standard) was spot-inoculated onto each plate, and visible growth was recorded after incubation at the optimal temperature for each strain (30 °C for Z022^T^, 25 °C for Z014^T^) for 7 days. Three independent biological replicates were performed. Considering that both of the strains are slightly orange-colored, the pigments were tested according to the method described by Biebl et al. [29].

Growth under anaerobic conditions (10% H_2_, 10% CO_2_, 80% N_2_) was determined after cultivation in an anaerobic chamber on modified MA, with or without 1% (*w*/*v*) KNO_3_ for at least 2 weeks at 30 °C. The modified MA supplemented with 1% (*v*/*v*) nitrate was used to test the reduction of nitrate with or without liquid paraffin at 30 °C for 7 days.

Oxidase activity was tested using the bioMérieux Oxidase Reagent kit (bioMérieux, Marcy-l’Étoile, France) according to the manufacturer’s instructions, and catalase activity was detected by measuring the production of oxygen bubbles in a 3% (*v*/*v*) aqueous hydrogen peroxide solution. The hydrolysis of starch, lipids, cellulose and alginate, and Tweens 20, 40, 60 and 80, was determined as previously described [14]. Susceptibility to antibiotics was investigated on MA for 7 days using filter-paper discs containing various antibiotics, as described previously [14] and according to procedures outlined by the Clinical and Laboratory Standards Institute (CLSI 2018).

The additional physiological and biochemical characteristics of cells of strains Z022^T^, Z014^T^, *P. filamentus* KCTC 23135^T^ and *P. atrinae* JCM 19202^T^ grown under the optimum conditions were assessed using the API 20E, API ZYM, and API 50CHB strips (bioMérieux, Marcy-l’Étoile, France) and Biolog GEN III MicroPlates according to the manufacturers’ recommendations, with the exception that the NaCl concentration was adjusted to 3% (*w*/*v*).

### 2.6. Chemotaxonomic Properties

For the determination of fatty acids, cells of strains Z022^T^, Z014^T^, *P. filamentus* KCTC 23135^T^ and *P. atrinae* JCM 19202^T^ were cultured on the MA at appropriate temperature and harvested after 3 days for growth. According to the standard protocol of MIDI (Sherlock Microbial Identification System, version 4.5), fatty acids were extracted, then methylated and analyzed by an Agilent 6890N gas chromatograph. Cellular fatty acids were identified using the TSBA40 database of the microbial identification system [30].

For polar lipids analysis, strains Z022^T^ and Z014^T^ were cultured in the modified MB at appropriate temperature and harvested after 3 days. Polar lipids were extracted from cells and separated via two-dimensional silica gel thin-layer chromatography (TLC). The total lipid materials were detected using molybdatophosphoric acid, and the functional groups were determined using spray reagents specific for particular functional groups. Polar lipids were determined using 2D TLC [31].

To analyze respiratory quinones, strains Z022^T^ and Z014^T^ grew on MB for 3 days were collected and freeze-dried. The procedures were carried out according to the methods described by Minnikin et al. [31], and quinone types were separated by HPLC [32].

## 3. Results and Discussion

### 3.1. 16S rRNA Gene Sequence and Phylogenetic Analysis

**Figure 1:**
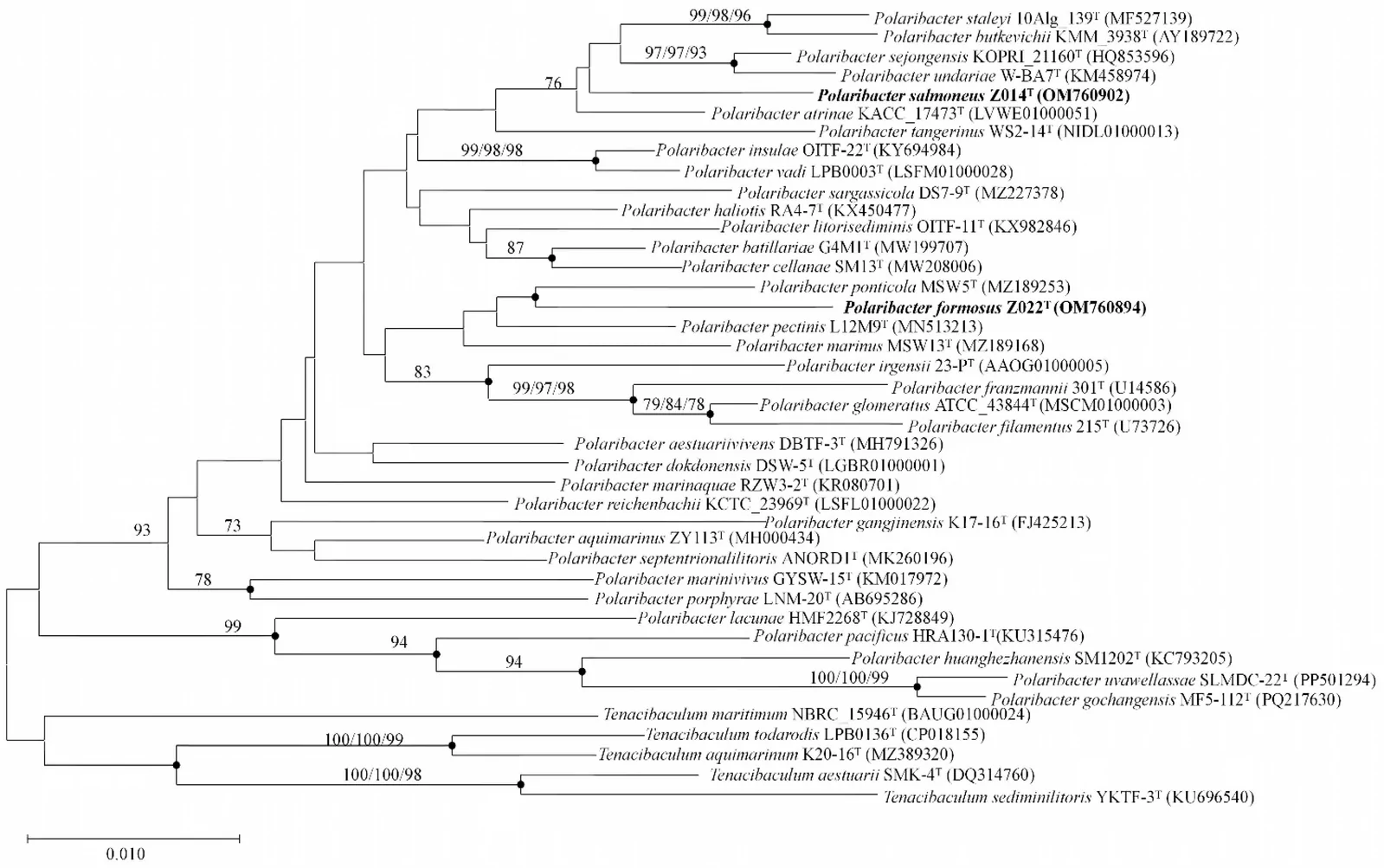
Neighbor-joining phylogenetic tree based on 16S rRNA gene sequences of strains Z022^T^, Z014^T^ and other closely related species. The strains characterized in this study are shown in bold type. Filled circles indicate branches that were recovered with all three methods (neighbor-joining, maximum-likelihood and maximum-parsimony). GenBank accession numbers of 16S rRNA gene sequences are given in parentheses. Percentage bootstrap values above 70% (1000 replicates) are shown at branch nodes. Strains of *Tenacibaculum* were used as the out-group. Bar, 0.01 substitutions per nucleotide position.

The complete 16S rRNA gene sequences of Z022^T^ (1520 bp) and Z014^T^ (1518 bp) were extracted from the genome assembly, which included the 16S rRNA gene sequences acquired from PCR and cloning. According to the comparison of 16S rRNA gene sequences, *P. ponticola* KACC 22340^T^ was the most similar strain to Z022^T^ (97.85%), and *P. atrinae* JCM 19202^T^ was the most similar strain to Z014^T^ (98.3%). The phylogenetic trees based on 16S rRNA genes (Figure 1) showed that strains Z022^T^ and Z014^T^ formed separate evolutionary branches within the genus *Polaribacter*, indicating that both strains were affiliated with the genus *Polaribacter*. Similar tree topologies were also shown in the 16S rRNA gene-based phylogenetic trees with the maximum-likelihood and maximum-parsimony algorithms (Supplementary Figures S1 and S2, available in the online Supplementary Materials).

### 3.2. Genomic Features and Phylogenomics

The draft genome of strain Z022^T^ was 3,497,066 bp and consisted of 49 contigs. The length of the contigs varied from 422 bp to 875,303 bp, with the N50 of 642,895 bp. The draft genome of strain Z022^T^ annotated with the NCBI Prokaryotic Genome Annotation Pipeline contained a total of 3105 genes, including 3054 protein-coding genes and 39 RNA-coding genes (34 tRNA genes, 1 complete 16s/23s rRNA gene, 3 ncRNA genes) and 12 pseudogenes. The genome size of strain Z014^T^ was 4,642,438 bp and consisted of 152 contigs. The length of the contigs of Z014^T^ varied from 3490 bp to 818,356 bp, with the N50 of 201,912 bp. The draft genome of strain Z014^T^ annotated with the NCBI Prokaryotic Genome Annotation Pipeline contained a total of 3853 genes, including 3781 protein-coding genes and 47 RNA-coding genes (42 tRNA genes, 1 complete 16s/23s rRNA gene, 3 ncRNA genes) and 25 pseudogenes. The sequencing depth was approximately 346× for strain Z022^T^ and 265× for strain Z014^T^. CheckM analysis showed that the completeness values of the genomes of Z022^T^ and Z014^T^ were 98.99% and 99.66%, and that the contamination values were 1.01% and 1.40%, respectively. The G + C contents of strains Z022^T^ and Z014^T^ are 28.3 mol% and 30.6 mol%.

**Figure 2:**
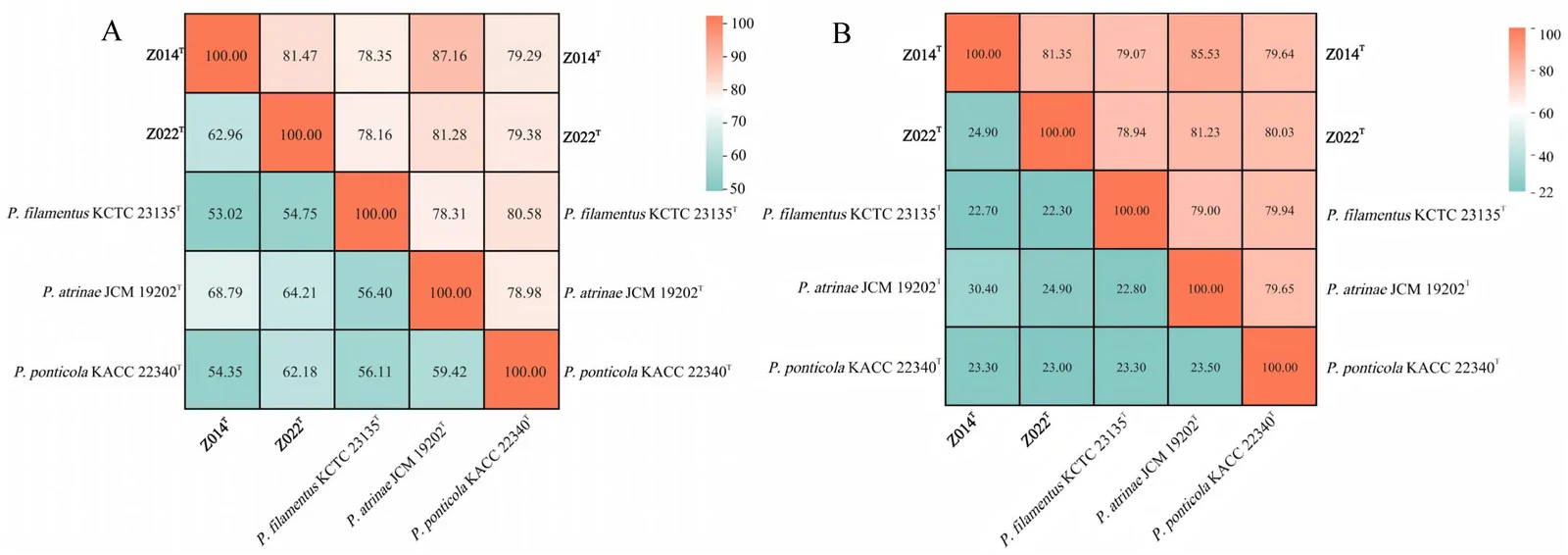
Heat maps showing POCP, AAI, dDDH and ANI values among Z022^T^, Z014^T^ and the reference strains. (**A**) Heat map based on POCP and AAI values; the half above the diagonal shows the AAI values and the half below shows the POCP values. (**B**) Heat map based on dDDH and ANI values; the half above the diagonal shows the ANI values and the half below shows the dDDH values.

**Figure 3:**
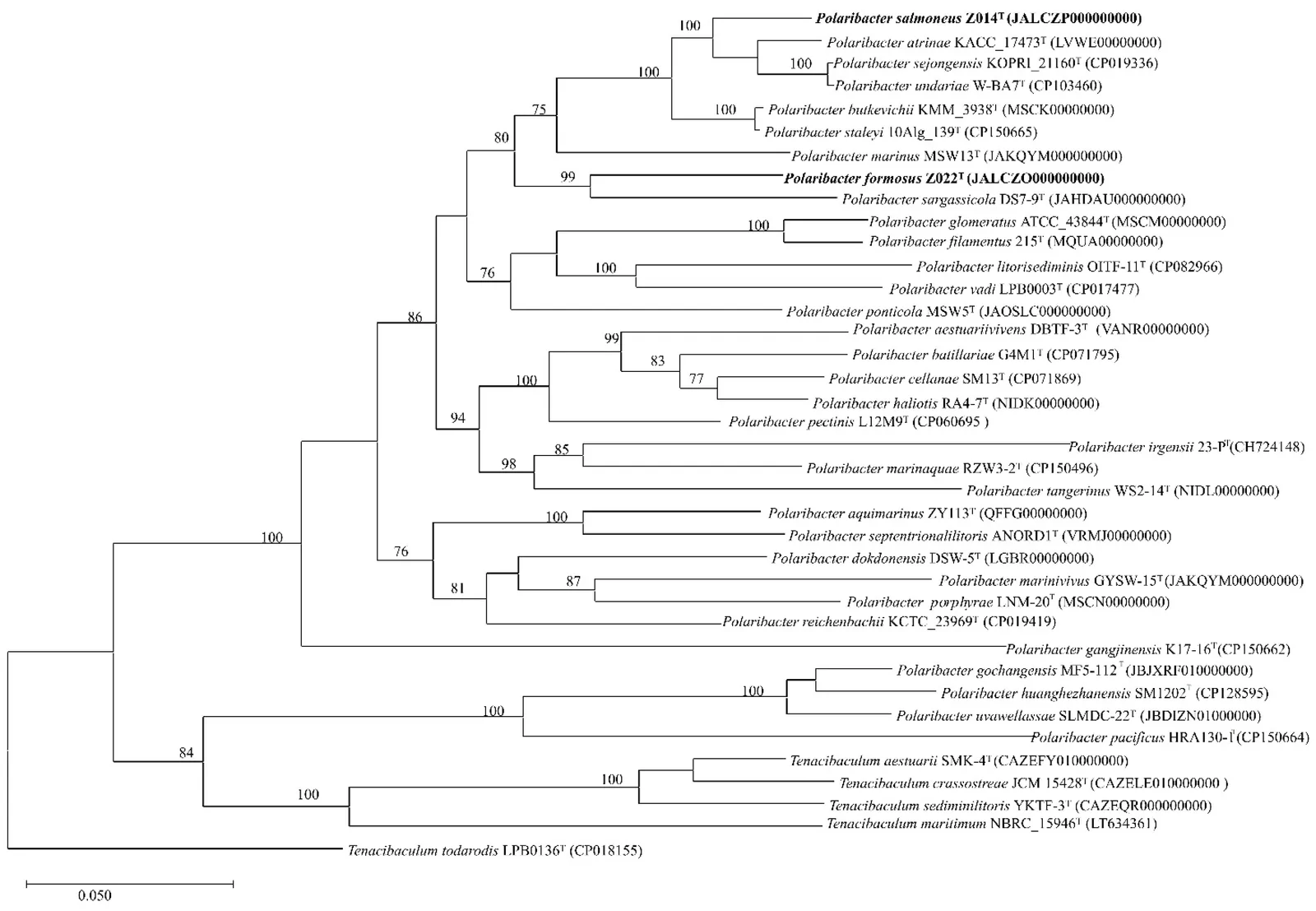
IQTree based on ribosomal protein sequences shows the phylogenetic relationships among Z022^T^, Z014^T^ and other species in *Polaribacter*. The strains characterized in this study are shown in bold type. GenBank accession numbers of whole-genome sequences are given in parentheses. Percentage bootstrap values above 70% (1000 replicates) are shown at branch nodes. Strains of *Tenacibaculum* were used as the out-group. Bar, 0.05 substitutions per nucleotide position.

The ANI, dDDH, AAI and POCP values were calculated among Z022^T^, Z014^T^ and the reference strains to analyze the genomic similarities (Figure 2). The ANI, dDDH, AAI and POCP values between Z022^T^ and Z014^T^ with the reference strains were 78.94–85.53%, 22.30–30.4%, 78.31–87.16% and 53.02–68.79%. The ANI, dDDH and AAI values were lower than the species delineation threshold (95%, 70% and 95%) [33,34], while the POCP values were higher than the genus delineation threshold (50%) [35]. The results indicated that Z022^T^ and Z014^T^ represented two novel species of the genus *Polaribacter*. The concatenated core gene phylogenetic tree (Figure 3), showing the evolutionary relationships of strain Z022^T^, Z014^T^ and other species within the genus *Polaribacter*, indicated that the two strains were affiliated with the genus *Polaribacter*, which was consistent with the result of 16S rRNA gene phylogenetic analysis.

### 3.3. Morphological, Physiological and Biochemical Characterizations

Cells of strains Z022^T^ and Z014^T^ were Gram-stain-negative and strictly aerobic. Colonies of Z022^T^ grown on MA were circular, smooth, light yellow and 2–3 mm in diameter; in addition, scanning electron microscopy (SEM, Supplementary Figure S3) pictures showed that Z022^T^ cells were long rod-shaped, approximately 1.57–3.86 μm in length and 0.22–0.37 μm in width. Colonies of Z014^T^ grown on MA were circular, smooth, convex, salmon-colored and 1–2 mm in diameter; the SEM pictures (Supplementary Figure S4) showed that Z014^T^ cells were short rod-shaped, approximately 0.68–1.33 μm in length and 0.37–0.49 μm in width. The optimal growth conditions for strains Z022^T^ and Z014^T^ were determined as follows: both strains grew optimally at 30 °C and 25 °C, with temperature ranges of 20–35 °C and 20–30 °C. The optimal pH values were 7.0 and 6.5, with growth ranges of 7.0–8.0 and 5.5–8.5, respectively. Additionally, both strains shared the same optimal NaCl concentration of 3% (*w*/*v*), with a growth range of 1.0–5.0% (*w*/*v*), respectively.

Both Z022^T^ and Z014^T^ could not grow under anaerobic conditions after two weeks of cultivation in an anaerobic chamber on an MA plate. Cells of Z022^T^ could glide on the semisolid agar while Z014^T^ remained stationary. Both Z022^T^ and Z014^T^ were non-motile in the liquid culture. The test for the reduction of nitrate was negative. Strains Z022^T^ and Z014^T^ exhibited strong degradation capabilities towards carbohydrates, such as starch and sodium alginate, which was consistent with the strong carbohydrate-degrading enzyme activities observed in other species of the genus *Polaribacter* [12]. In addition, both of Z022^T^ and Z014^T^ could hydrolyze Tweens 20, 40, 60, 80 and had catalase activity, while the casein and cellulose were not hydrolyzed by either of the strains. Z014^T^ had oxidase activity, which is consistent with the most closely related strain *P. atrinae* JCM 19202^T^ [6], while Z022^T^ lacked oxidase activity, which is different from the reference strain *P. ponticola* KACC 22340^T^ [10]. The inhibition-zone diameters of strains Z022^T^ and Z014^T^ are presented in Supplementary Table S2. According to the criteria, both strains Z022^T^ and Z014^T^ were found to be susceptible to erythromycin (15 µg), ampicillin (10 µg), penicillin (10 µg), chloramphenicol (30 µg), rifampin (5 µg), cefotaxime sodium (30 µg), ofloxacin (5 µg), lincomycin (2 µg), carbenicillin (100 µg) and clarithromycin (15 µg) but resistant to tobramycin (10 µg), neomycin (30 µg), gentamycin (10 µg), streptomycin (10 µg), kanamycin (30 µg) and polymyxin B (30 µg). In addition, strain Z014^T^ could be resistant to tetracycline (30 µg) and norfloxacin (30 µg).

The phenotypic characteristics, determined by the API ZYM, API 20E, API 50CHB identification systems (BioMérieux) and Biolog GEN IIIMicroPlates, of strains Z022^T^, Z014^T^ and the reference strains *P. atrinae* JCM 19202^T^ and *P. filamentus* 215^T^ are listed in Supplementary Table S3. Z022^T^, Z014^T^ and the reference strains showed some common enzymatic activities, such as the positive reactions with alkaline phosphatase, leucine arylamidase, valine arylamidase, cystine arylamidase, acid phosphatase and naphthol-AS-BI-phosphohydrolase, and negative reactions with *β*-glucuronidase and *α*-mannosidase. The detailed different physiological and biochemical characteristics between those strains are summarized in Table 1.

Cadmium is one of the most toxic heavy metals, and its bioaccumulation and potential biomagnification have been documented in Antarctic marine organisms [36]. Strain Z014^T^ showed visible growth on MA plates supplemented with CdCl_2_ at concentrations up to 0.3 mM under the tested conditions, while Z022^T^ was sensitive to cadmium chloride and did not grow at ≥0.1 mM cadmium chloride (Supplementary Table S4). According to the KEGG and RAST annotations of Z014^T^, we found some key genes related to the cadmium detoxification. The *cadA* gene could encode the cadmium-translocating P-type ATPase, which could pump low concentrations of the cadmium ions out of the cell [37]. The *czcA* gene could encode the heavy metal efflux RND transporter, which could also pump the cadmium ions out of the cell, especially in case of the excessive accumulation of cadmium [38]. In addition, we also found some antioxidant genes, such as the *msrA*, *msrB* and *katG*, which could repair the proteins damaged caused by reactive oxygen species generated by heavy metals such as the cadmium ions [39,40]. Genome mining revealed that those genes may contribute to the cadmium-tolerant ability of Z014^T^, and their functional role in cadmium tolerance remains to be experimentally verified. Carotenoid pigments were present in both strains.

### 3.4. Pan-Genome Analysis of the Genus Polaribacter

**Figure 4:**
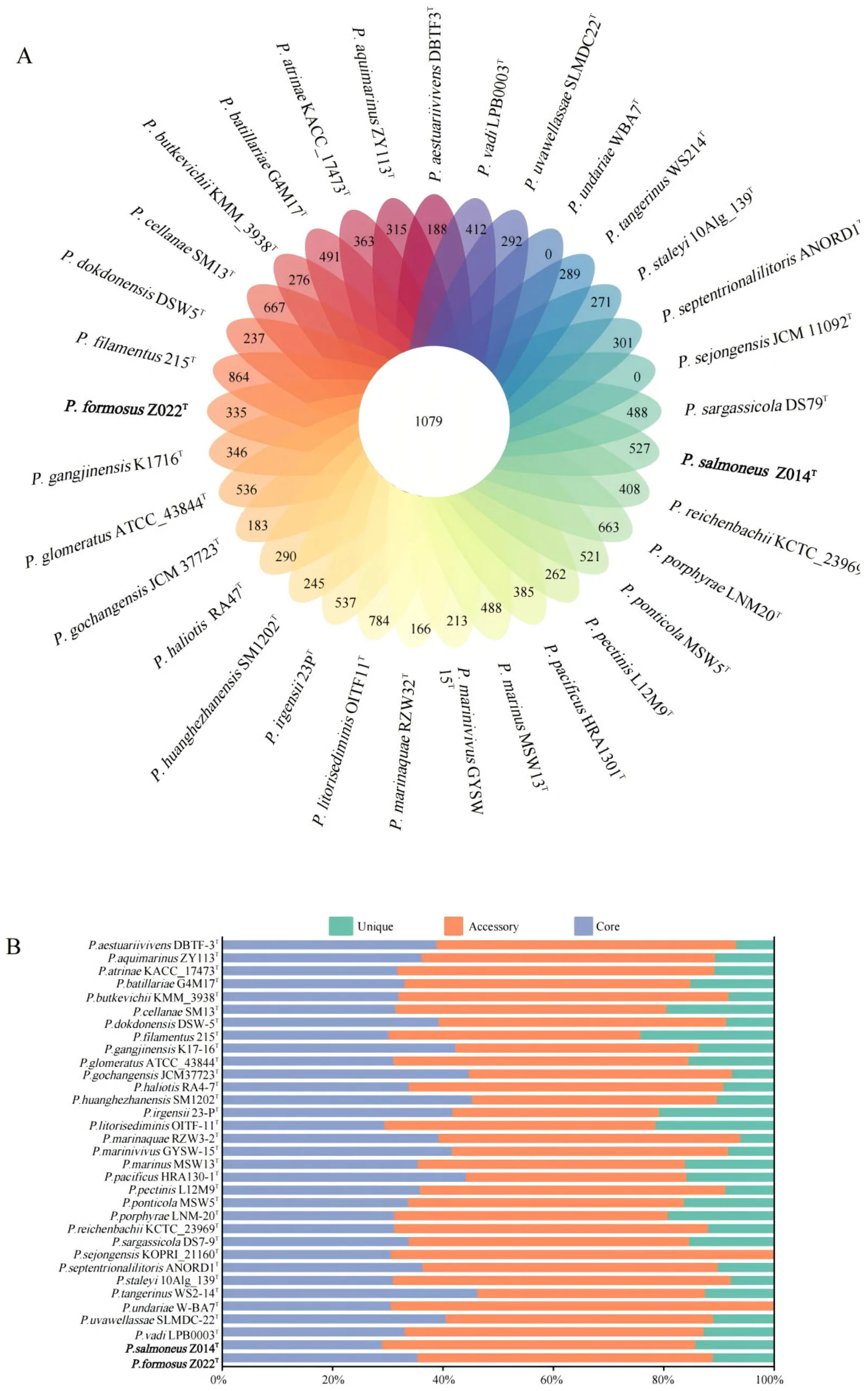
Comparisons of orthologous protein groups in the genera *Polaribacter*. (**A**) Venn diagram displaying the numbers of core gene families and unique genes for each of the 33 members. (**B**) Percentage of core, accessory and unique genes in each of the 33 genomes. Strains researched in this article are indicated in bold.

Pan-genome analysis was conducted with the BPGA to identify orthologous groups among Z022^T^, Z014^T^ and other strains within the genus *Polaribacter*. Comparative analysis based on the orthologous proteins revealed that 1079 core genes were shared among the 33 strains (Figure 4A). The percentages of the core genes in each genome ranged from 29.01% to 46.29% (Figure 4B). The core genes contributed to the amino acid metabolism, carbohydrate metabolism, nucleotide metabolism, translation, energy metabolism, replication and repair, which comprise the basic metabolism for survival (Supplementary Figure S5). These genes also conferred the strains with the ability to adapt to oligotrophic marine environments. Among the 33 strains, Z014^T^ had the lowest proportion of core genes (29.01%), followed by *P. litorisediminis* OITF-11^T^ (29.45%) and *P. filamentus* 215^T^ (30.17%). The proportion of the unique genes varied from 0% (*P. undariae* W-BA7^T^, *P. sejongensis* KOPRI_21160^T^) to 24.16% (*P. filamentus* 215^T^). Results of the core pan-genome prediction model showed that the pan-genome increased with the addition of new strains and was far from saturation, which indicated that the pan-genome of the genus *Polaribacter* was open (Supplementary Figure S6).

### 3.5. Environments and Biogeographic Distribution

**Figure 5:**
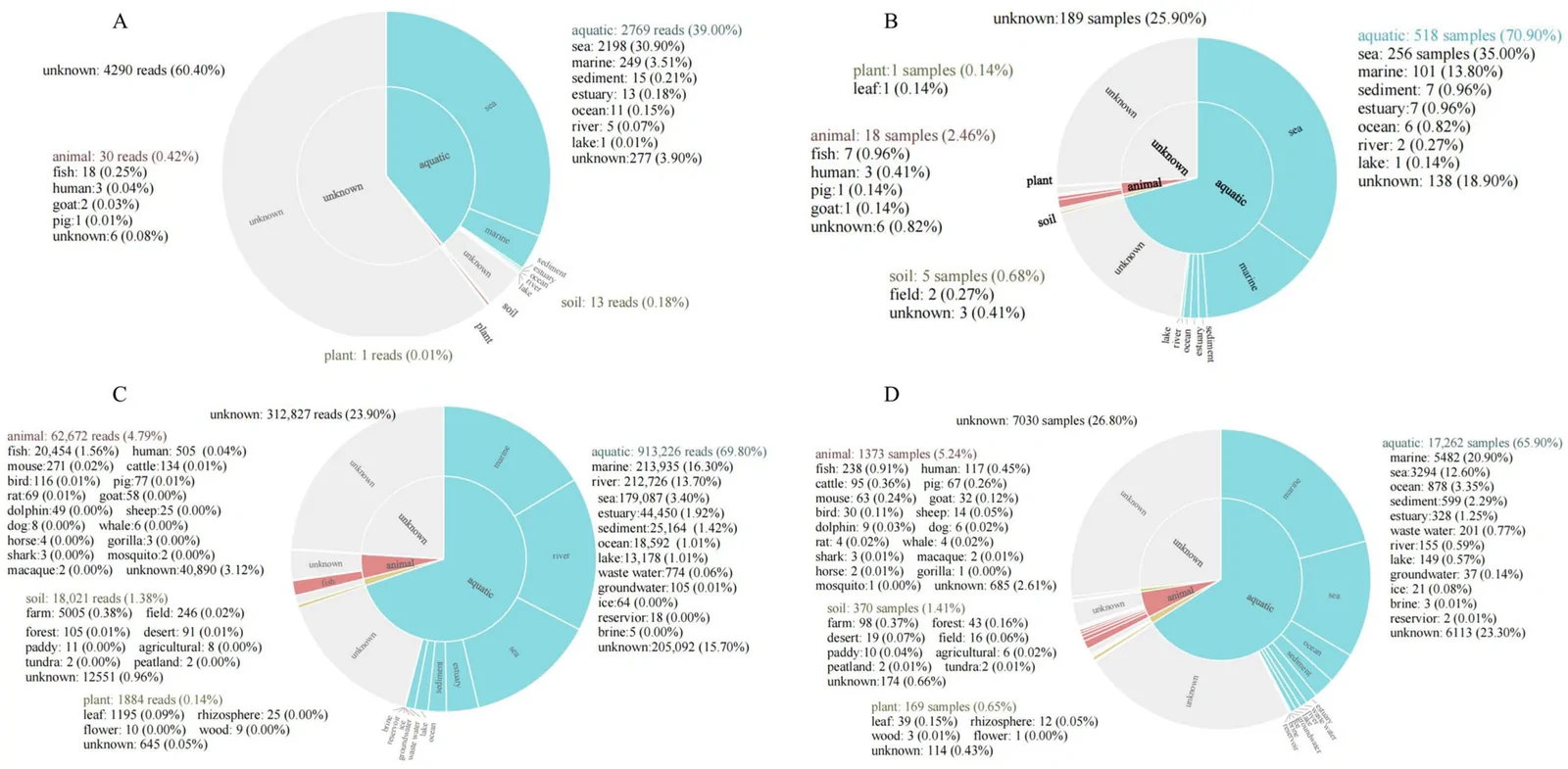
Biogeographic distribution analysis of the strains Z022^T^ and Z014^T^ based on the Microbe Atlas Project (MAP) database and pipeline. (**A**) Number of sequencing reads mapping to the representative OTU sequence of Z014^T^, per habitat and sub-habitat. (**B**) Number of samples containing the representative OTU sequence of Z014^T^, per habitat and sub-habitat. (**C**) Number of sequencing reads mapping to the representative OTU sequence of Z022^T^, per habitat and sub-habitat. (**D**) Number of samples containing the representative OTU sequence of Z022^T^, per habitat and sub-habitat.

The global distribution of the species Z014^T^ and Z022^T^ was analyzed with the MAP database (Figure 5). Sequences with 99% similarity to Z014^T^ were found in 731 samples from 177 projects. The majority of sequences with 99% similarity to Z014^T^ were predominantly found in aquatic habitats (70.9%), which is consistent with the situation that most of the strains were isolated from the marine environments. There were also relatively fewer sequences found in animals (2.46%), soil (0.68%) and plants (0.14%). Sequences with 97% similarity to Z022^T^ were found in 26,204 samples from 2782 projects; aquatic habitats were also the predominant habitats (65.90%). The quantitative analysis of the database sequencing reads mapping to the representative OTU sequence showed that the highest number of related reads to Z014^T^ and Z022^T^ were also from aquatic environments (39.00% and 69.8%).

### 3.6. Metabolic Analysis of the Genus Polaribacter

**Figure 6:**
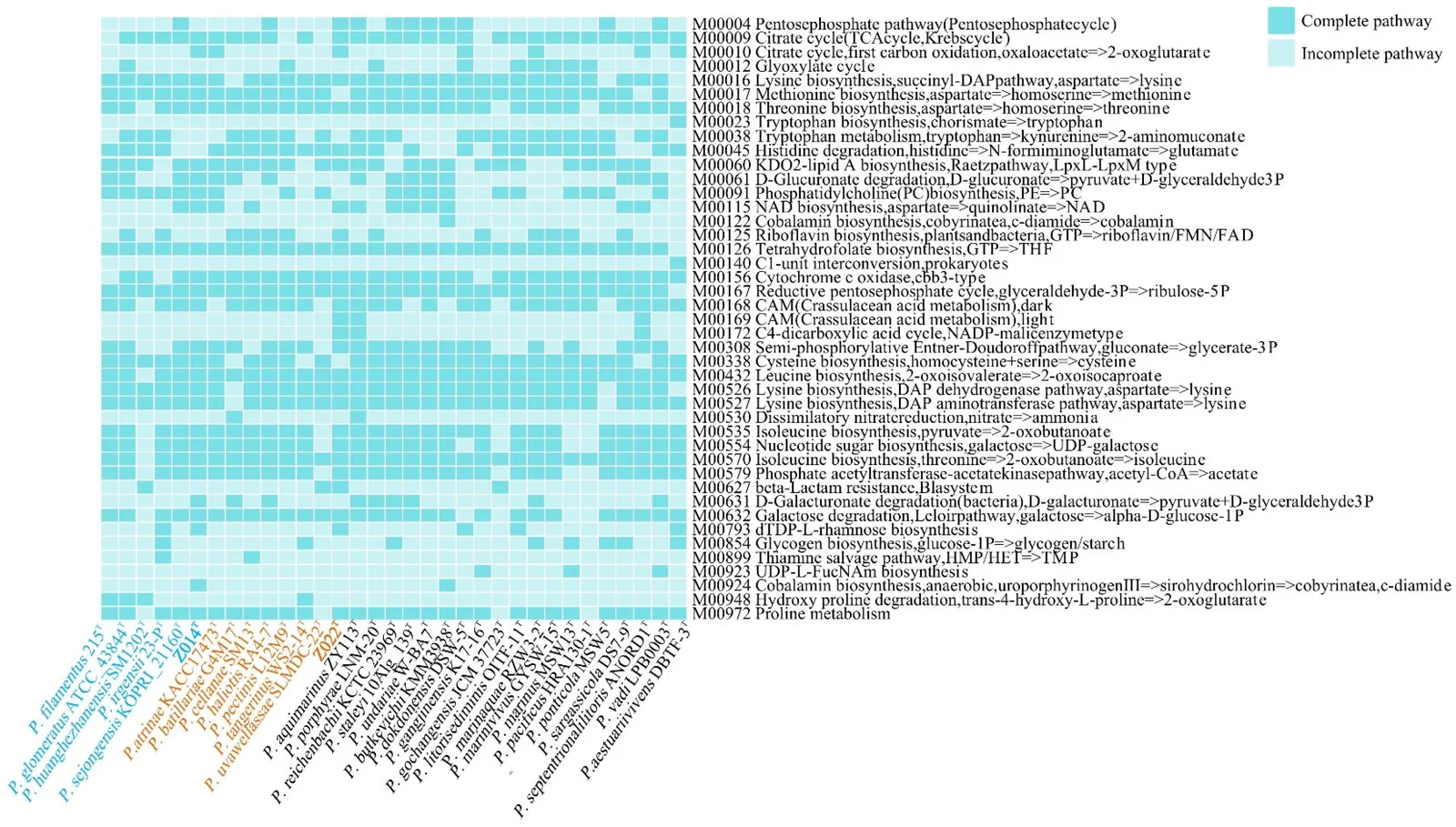
Heat map of complete and incomplete metabolic pathways in the genomes of the 33 species of the genus *Polaribacter*.

The results of the metabolic pathway analysis with KEGG’s BlastKOALA of the genus *Polaribacter* are shown in Figure 6 and Supplementary Table S5. Strains colored blue were isolated from polar regions (e.g., Z014^T^); the strains colored brown were isolated from the marine animals (e.g., Z022^T^). The results showed that all strains within the genus *Polaribacter* possessed the complete central carbohydrate metabolism pathways, such as glycolysis (M00001, M00002), gluconeogenesis (M00003) and the citrate cycle (M00011, M00307) (Supplementary Table S5). Some amino acid metabolisms (including the complete synthesis and degradation), cofactor synthesis pathways and fatty acid biosynthesis pathways were also present in all of the strains (Supplementary Table S5). Those relatively complete basic metabolism pathways contributed to the better adaptation of strains within the genus *Polaribacter* to oligotrophic marine environments.

A higher proportion of strains isolated from the polar regions, such as Z014^T^, possessed complete phosphatidylcholine biosynthesis pathways (M00091) compared with the strains isolated from other environments. The polar-isolated strains could modulate the PC/PE ratio within their cytoplasmic membranes. This headgroup modification altered the lipid bilayer’s biophysical properties, thereby maintaining optimal membrane fluidity and structural integrity under sub-zero temperatures, which is a critical adaptive strategy for surviving in polar environments [41,42]. The KDO_2_-lipid A biosynthesis pathway (M00060) was also present in the polar region isolated strains. KDO_2_-lipid A is one of the core components of lipopolysaccharide in the outer membrane of Gram-negative bacteria. For strains under low-temperature polar environments, *LpxP* would be induced to incorporate unsaturated palmitoleic acid instead of lauric acid into the lipid A precursor, which could lower the melting point and maintain the membrane fluidity of the strains [43]. Similar results were found in the Arctic water-isolated *Polaribacter* sp. SM1127: the structural heterogeneity in lipid A could enable the bacterium to fine-tune the cold-related environmental stress [44]. Half of the strains isolated from the polar regions possessed the complete hydroxyproline degradation pathway (M00948). Hydroxyproline was enriched in the glycoproteins within algal cell walls, such as the diatoms inhabited in the polar sea ice [45]. The degradation and utilization of proline and hydroxyproline can act as bioavailable carbon and nitrogen substrates for the strains isolated from the cold region under oligotrophic conditions, which may represent an adaptive strategy for bacteria inhabitinged the ice-alga-rich polar environment [46].

Z022^T^ was the sole strain which possessed the dTDP-L-rhamnose biosynthesis pathway (M00793). dTDP-L-rhamnose was the precursor required for constructing lipopolysaccharide and extracellular polysaccharides in Gram-negative bacteria [47]. The ability to synthesize L-rhamnose facilitated the assembly of intact outer-membrane LPS, conferring resistance to digestive enzymes and osmotic stress within the abalone digestive tract [48]. In addition, Z022^T^ was one of the few strains to possess the complete beta-lactam resistance pathway (M00627). This module encoded the regulatory and structural components required for inducible β-lactamase production, which could enable bacteria to hydrolyze the β-lactam antimicrobial compounds produced by the other microorganisms colonized in the intestinal tract [49]. Those two complete pathways improved the survival and colonization capacity of Z022^T^ within the abalone visceral niche.

### 3.7. Chemotaxonomic Characteristics

The predominant cellular fatty acids of strain Z022^T^ were iso-C_15:0_ (43.9%) and iso-C_15:0_ 3-OH (16.8%). The predominant cellular fatty acids of strain Z014^T^ were iso-C_15:0_ 3-OH (18.49%), iso-C_15:0_ (11.73%) and C_15:0_ 3-OH (10.36). Iso-C_15:0_ and iso-C_15:0_ 3-OH were the most predominant fatty acids among these two novel strains and the three reference strains. The major predominant fatty acids of Z014^T^ and the reference strain *P. sejongensis* JCM 18092^T^ were the same. The detailed fatty acid compositions of strains Z022^T^, Z014^T^ and the reference strains are shown in Supplementary Table S6. The major polar lipids of the two strains are shown in Supplementary Figure S7. The major polar lipids of strain Z022^T^ were phosphatidylethanolamine (PE), aminolipid (AL) and three unidentified lipids (L1-3). The major polar lipids of strain Z014^T^ were aminolipid (AL), aminoglycolipid (AGL) and four unidentified lipids (L1-4). Menaquinone-6 was the sole respiratory quinone of both Z022^T^ and Z014^T^, which was the same with the other species within the genus *Polaribacter*.

## 4. Conclusions

According to all these results of phenotypic, biochemical and physiological analyses, together with the phylogenetic differences, strains Z022^T^ and Z014^T^ could be assigned to the genus *Polaribacter* within the family *Flavobacteriaceae*. As representing two novel species, *Polaribacter formosus* sp. nov. and *Polaribacter salmoneus* sp. nov. are proposed, with Z022^T^ and Z014^T^ as the type strains, respectively.

### 4.1. Description of Polaribacter formosus sp. nov.

*Polaribacter formosus* (for.mo’sus. L. masc. adj. *formosus*, beautiful, beautifully formed).

Cells are Gram-stain-negative, strictly aerobic and long rod-shaped. Colonies grown on MA plates were circular, smooth, light yellow and 2–3 mm in diameter. Optimal growth occurred at 30 °C (range 20–35 °C) and pH 7.0 (range 7.0–8.0) with 3% (*w*/*v*) NaCl (range 1.0–5.0%). Cells could glide on the semisolid agar. Cells could hydrolyze starch, alginate and Tweens 20, 40, 60, 80, and had catalase activity, while casein and cellulose were not hydrolyzed. The test for the reduction of nitrate was negative. Cells can produce alkaline phosphatase, esterase (C4), esterase lipase (C8), leucine arylamidase, valine arylamidase, cystine arylamidase, trypsin, *a*-chymotrypsin, acid phosphatase, N-acetyl-*β*-glucosaminidase, *β*-fucosidase and naphthol-AS-BI-phosphohydrolase. Positive for Voges–Proskauer reaction. Acids are produced from d-ribose, l-sorbose, d-tagatose and potassium 5-ketogluconate, and weakly from potassium 2-keto-gluconate, d-arabinose, d-xylose, l-xylose, d-fructose, d-mannitol, arbulin, aesculin ferric citrate, d-raffinose and d-lyxose. Cells can oxidize d-mannose, glucuronamide, *β*-hydroxy-d, l-butyric acid and formic acid. The major respiratory quinone is menaquinone-6. The main polar lipids contain phosphatidylethanolamine (PE), aminolipid (AL) and three unidentified lipids (L1-3). The dominant fatty acids are iso-C_15:0_ (43.9%) and iso-C_15:0_ 3-OH (16.8%). The DNA G + C content is 28.3 mol%.

The type strain, Z022^T^ (=MCCC 1H00505^T^ = KCTC 92009^T^), was isolated from the visceral of abalone, *Haliotis discus hannai*, in Weihai, China. The GenBank accession numbers of strain Z022^T^ for the 16S rRNA gene and genome sequences are OM760894 and JALCZO000000000, respectively.

### 4.2. Description of Polaribacter salmoneus sp. nov.

*Polaribacter salmoneus* (sal.mo’ne.us. N.L. masc. adj. *salmoneus*, salmon-colored, because the colonies grown on the MA were salmon-colored).

Cells are Gram-stain-negative, strictly aerobic and short rod-shaped. Colonies grown on MA plates were circular, smooth, convex, salmon-colored and 1–2 mm in diameter. Optimal growth occurred at 25 °C (range 20–30 °C) and pH 6.5 (range 5.5–8.5) with 3% (*w*/*v*) NaCl (range 1.0–5.0%). Cells could hydrolyze starch and alginate and Tweens 20, 40, 60, 80, and had catalase and oxidase activity, while the casein and cellulose were not hydrolyzed. The test for the reduction of nitrate was negative. Cells can produce alkaline phosphatase, esterase (C4), esterase lipase (C8), leucine arylamidase, valine arylamidase, cystine arylamidase, trypsin, *a*-chymotrypsin, acid phosphatase, naphthol-AS-BI-phosphohydrolase and *α*-glucosidase. Positive for o-nitrophenyl-*β*-D-galactopyranoside and Voges–Proskauer reaction. Acids are produced from d-xylose, d-galactose, d-glucose, d-fructose, d-mannose, d-mannitol, methyl-*α-*d-mannopyranoside, amygdaline, arbulin, aesculin ferric citrate, d-cellobiose, d-maltose, d-lactose, d-melibiose, d-melezitose, starch, glycogen, gentiobiose, l-fucose and potassium 5-keto-gluconate. Cells can oxidize d-salicin, *α*-d-glucose, myo-inositol, d-fructose-6-PO_4_, d-galacturonic acid, l-galactonic acid lactone, d-glucuronic acid, glucuronamide, methyl pyruvate, l-lactic acid, *α*-keto-glutaric acid, d-malic acid, l-malic acid, *α*-hydroxy-butyric acid, *β*-hydroxy-d, l-butyric acid, acetoacetic acid and propionic acid. The major respiratory quinone is menaquinone-6. The main polar lipids contain aminolipid (AL), aminoglycolipid (AGL) and four unidentified lipids (L1-4). The dominant fatty acids are iso-C_15:0_ 3-OH (18.49%), iso-C_15:0_ (11.73%) and C_15:0_ 3-OH (10.36%). The DNA G + C content is 30.6 mol%.

The type strain, Z014^T^ (=MCCC 1H00504^T^ = KCTC 92010^T^), was isolated from sediment samples collected from the Antarctica Great Wall station. The GenBank accession numbers of strain Z014^T^ for the 16S rRNA gene and genome sequences are OM760902 and JALCZP000000000, respectively.

## Figures and Tables

**Table 1:** Differential characteristics between strain Z022^T^, Z014^T^ and related species.

Characteristic	1	2	3	4 ^a^	5
Isolation source	Viscera of abalone	Sediment in Antarctica	Intestine of comb pen shell	Seawater of Yellow Sea	Seawater in Arctic
Colony color on MA	Light yellow	Salmon	Yellow	Yellow	Salmon
Growth range (optimum) of:					
Temperature (°C)	20–35 (30)	20–30 (25)	4–30 (20)	15–25 (25)	4–19 (4)
NaCl (%)	1.0–5.0 (3)	1.0–5.0 (3)	1–6 (2)	1–5 (2)	1–5
pH	7.0–8.0 (7.0)	5.5–8.5 (6.5)	5–9 (7)	5–9 (7–8)	6.0–8.5
Hydrolysis of:					
Starch	+	+	+	−	w
DNA	−	+	+	NA	NA
Tween 20	+	+	+	−	NA
casein	−	−	+	−	−
Enzyme activity:					
esterase (C4)	+	+	w	+	−
esterase lipase (C8)	+	+	−	+	−
trypsin	+	+	+	−	+
*a*-chymotrypsin	+	+	−	−	−
*α*-galactosidase	−	−	+	+	−
*β*-galactosidase	−	−	−	+	−
*α*-glucosidase	−	+	−	−	−
N-acetyl-*β*-glucosaminidase	+	−	+	−	−
*β*-fucosidase	+	−	−	−	−
oxidase	−	+	+	+	−
Nitrate reduction	−	−	+	−	−
citrate utilization	−	−	+	NA	+
Voges–Proskauer reaction	+	+	w	NA	−
gliding	+	−	−	−	NA
Genome size (Mb)	3.5	4.6	4.0	3.6	4.3
GC content (%)	28.3	30.6	30.5	29.1	31.4

Strains: 1, Z022^T^; 2, Z014^T^; 3, *Polaribacter atrinae* JCM 19202^T^; 4, *Polaribacter ponticola* KACC 22340^T^; 5, *Polaribacter filamentus* 215^T^. All data were from this study unless indicated otherwise. +, positive; −, negative; w, weakly positive; NA, no data available. a: Data from Baek et al. [10]. Values in parentheses represent optimal growth conditions.

## Data Availability

The GenBank accession numbers for the 16S rRNA gene sequences of *Polaribacter formosus* Z022^T^ and *Polaribacter salmoneus* Z014^T^ are OM760894 and OM760902, respectively. The genomes of *Polaribacter formosus* Z022^T^ and *Polaribacter salmoneus* Z014^T^ have been deposited in GenBank under the accession numbers JALCZO000000000 and JALCZP000000000, respectively.

## Supplementary Materials

The following supporting information can be downloaded at: https://www.mdpi.com/article/10.3390/microorganisms14102227/s1, Figure S1: Maximum-likelihood phylogenetic tree based on 16S rRNA gene sequences of strains Z022^T^, Z014^T^ and other closely related species. GenBank accession numbers of 16S rRNA gene sequences are given in parentheses. Percentages bootstrap values above 70% (1000 replicates) are shown at branch nodes. Strains of *Tenacibaculum* were used as the out-group. Bar, 0.02 substitutions per nucleotide position. Figure S2:Maximum-parsimony phylogenetic tree based on 16S rRNA gene sequences of strains Z022^T^, Z014^T^ and other closely related species. GenBank accession numbers of 16S rRNA gene sequences are given in parentheses. Percentages bootstrap values above 70% (1000 replicates) are shown at branch nodes. Strains of *Tenacibaculum* were used as the out-group. Figure S3: Scanning electron micrograph of cells of strain Z022^T^. Bar, 2 μm. Figure S4: Scanning electron micrograph of cells of strain Z014^T^. Bar, 3 μm. Figure S5: The distribution of core genes, accessory genes and unique genes to different metabolic pathways in the genus *Polaribacter*. Figure S6: Pan-and core-genome of the genus *Polaribacter*. Triangle are the average of values obtained for the different strain combinations. Pan-genome power-fit curve equation: f(x) = 2991.08x^0.5650^, core-genome exponential curve equation: f1(x) = 1902.26e^−0.0248x^. Figure S7: Two-dimensional TLC plate image of the total polar lipids of strain Z022^T^ (a) and Z014^T^ (b). PE, phosphatidylethanolamine; AL, unidentified aminolipid; AGL, aminoglycolipid; L, unidentified lipid. Table S1: Genomic information of the species belonging to genus *Polaribacter* researched in this article. Table S2: The inhibition-zone diameters of strains Z022^T^ and Z014^T^. The results are the mean of three replicates. Table S3: List of phenotypic response to API 20E, API ZYM and API 50CHB identification systems (bioMérieux) and Biolog GEN III MicroPlates. Strains: 1, Z022^T^; 2, Z014^T^; 3, *Polaribacter filamentus* 215^T^; 4, *Polaribacter atrinae* JCM 19202^T^. All data are from this study. +, Positive; –, negative; w, weakly positive. Table S4: Cadmium resistance of strains Z022^T^ and Z014^T^. Growth was assessed on marine agar supplemented with different concentrations of CdCl_2_ (0–0.5 mM). Symbols: +++, good growth; ++, moderate growth; +, weak growth; −, no growth. Table S5: Metabolic pathway analysis with KEGG’s BlastKOALA of the genus *Polaribacter*. Table S6: Cellular fatty acid composition (%) of strain Z022^T^, Z014^T^ and the closely related type strains.

## Abbreviations

ANI, Average Nucleotide Identity; AAI, Average Amino Acid Identity; POCP, Percentage of Conserved Proteins; dDDH, Digital DNA-DNA Hybridization; GTDB, Genome Taxonomy Database; HPLC, High-Performance Liquid Chromatography; MA, Marine Agar 2216; MB, Marine Broth 2216; MCCC, Marine Culture Collection of China; KCTC, The Korean Collection for Type Cultures; MEGA, Molecular Evolutionary Genetics Analysis; MIDI, Microbial Identification System; NCBI, National Center of Biotechnology Information; RAST, Rapid Annotation using Subsystem Technology; TLC, Thin-Layer Chromatography; PCR, polymerase chain reaction.
