# Supplementary material for: Characterization of Polaribacter formosus sp. nov. and Polaribacter salmoneus sp. nov. with Comparative Genomic Analysis of the Genus Polaribacter
Source: Microorganisms. 2026 Oct 2;14(10):2227. doi: 10.3390/microorganisms14102227 (PMC13707730; doi:10.3390/microorganisms14102227)
Supplement: Supplementary file 1 [file microorganisms-14-02227-s001.zip › Supplementary Materials/Supplementary Materials.pdf]

Supplementary Materials

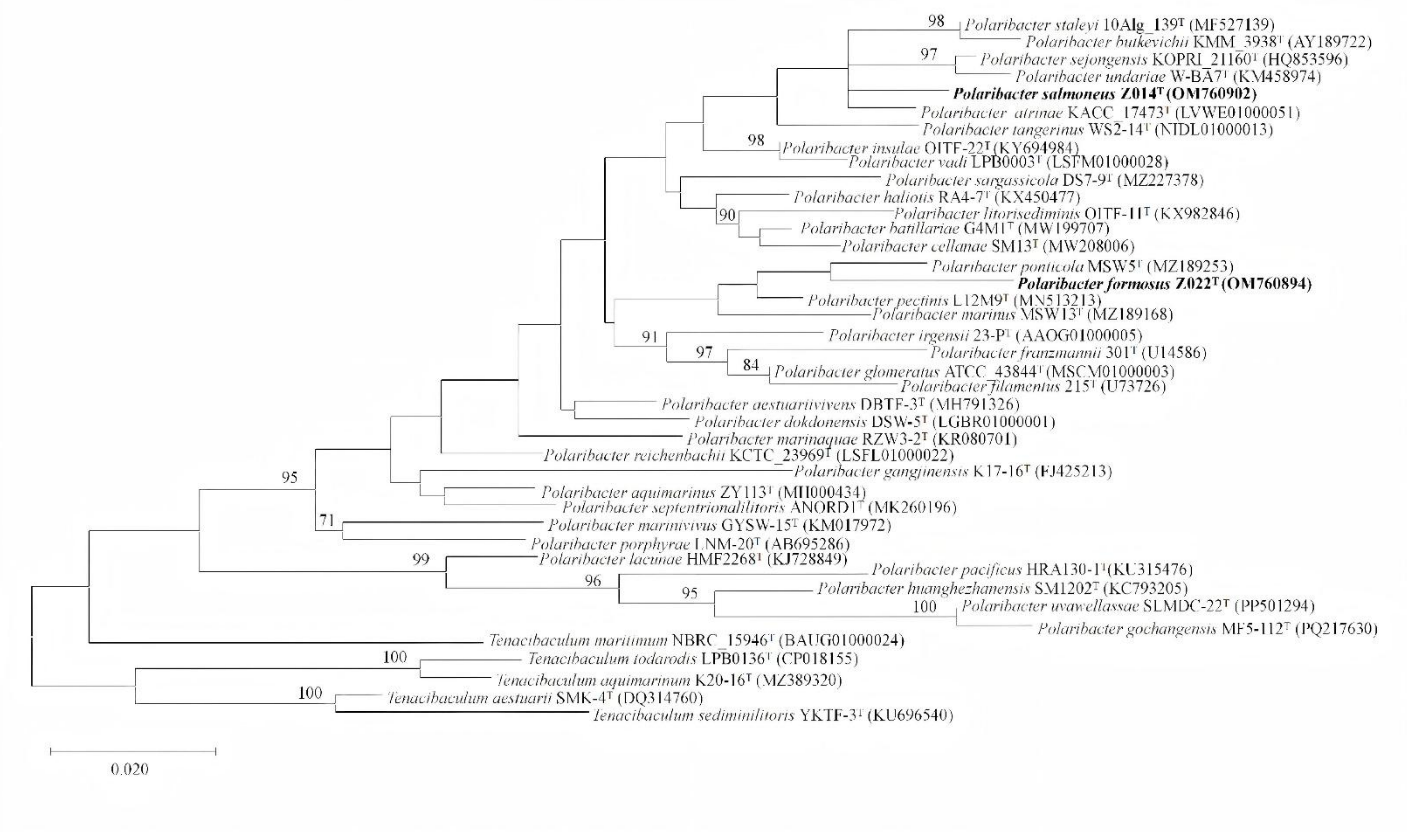

Figure S1. Maximum-likelihood phylogenetic tree based on 16S rRNA gene sequences of strains Z022^{T}, Z014^{T} and other closely related species. GenBank accession numbers of 16S rRNA gene sequences are given in parentheses. Percentages bootstrap values above 70% (1000 replicates) are shown at branch nodes. Strains of *Tenacibaculum* were used as the out-group. Bar, 0.02 substitutions per nucleotide position.

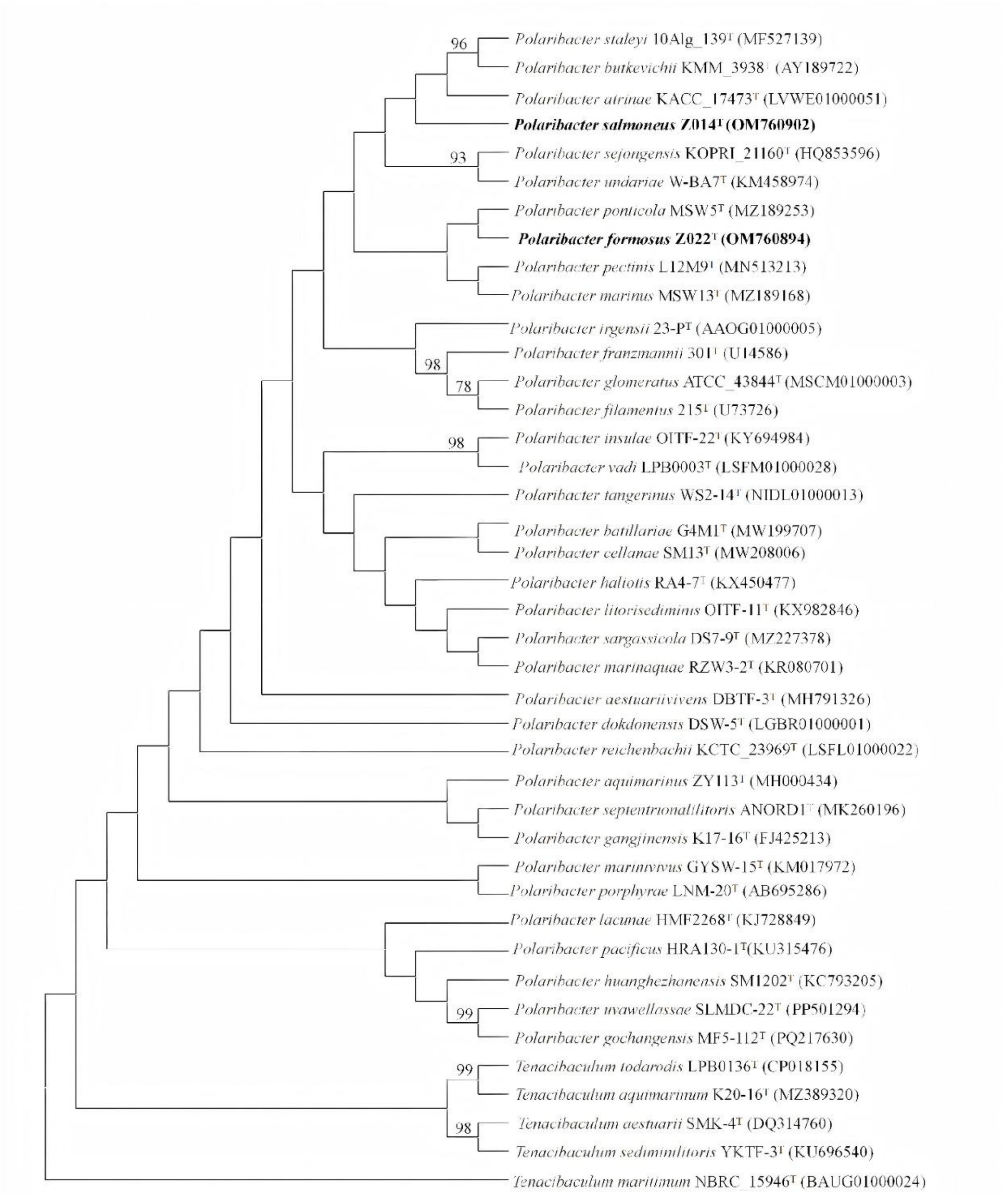

Figure S2. Maximum-parsimony phylogenetic tree based on 16S rRNA gene sequences of strains Z022^{T}, Z014^{T} and other closely related species. GenBank accession numbers of 16S rRNA gene sequences are given in parentheses. Percentages bootstrap values above 70% (1000 replicates) are shown at branch nodes. Strains of *Tenacibaculum* were used as the out-group.

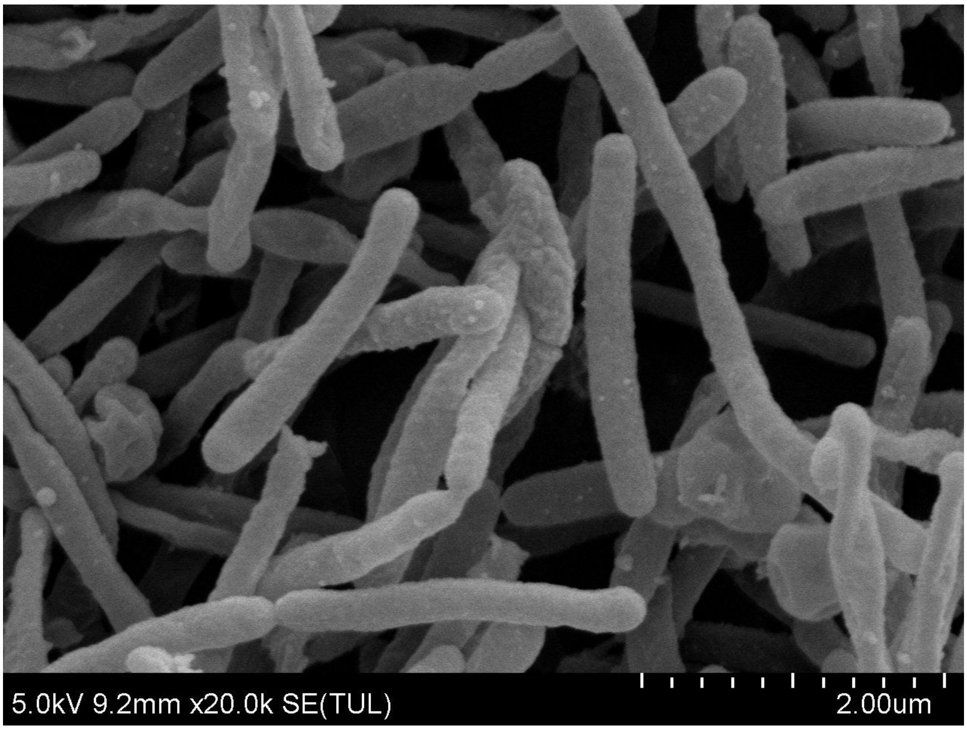

Figure S3. Scanning electron micrograph of cells of strain Z022^{T}. Bar, 2 µm.

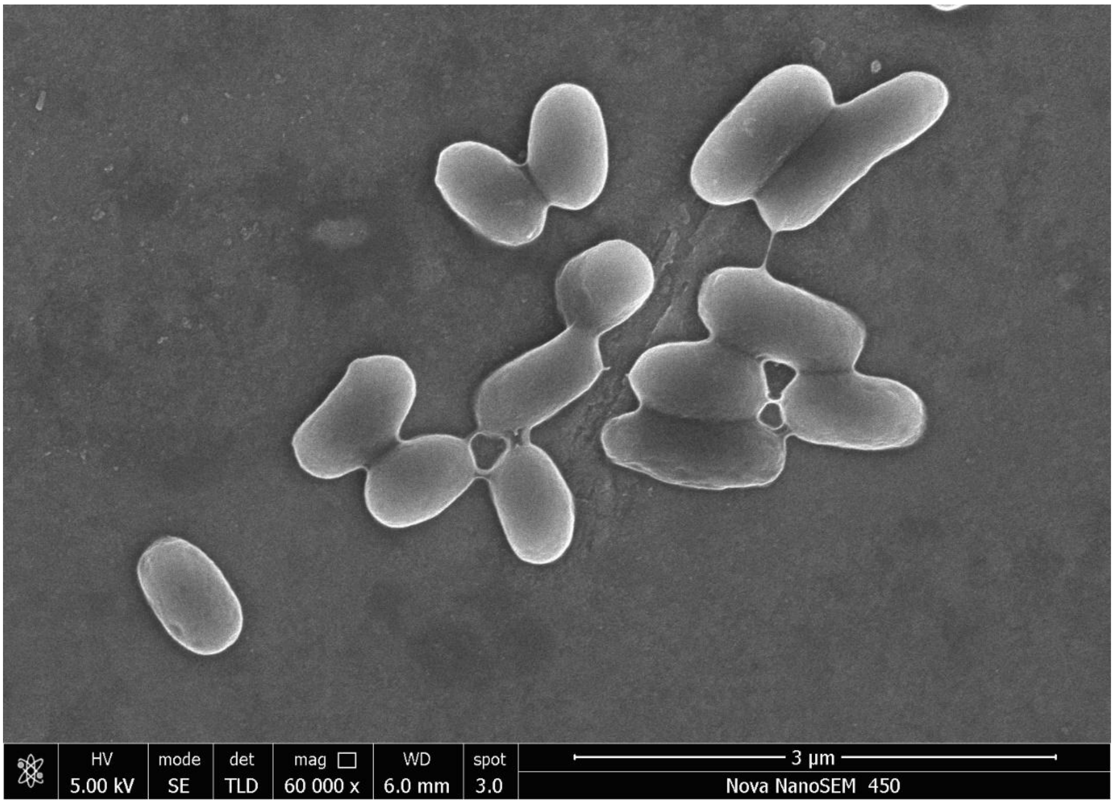

Figure S4. Scanning electron micrograph of cells of strain Z014^{T}. Bar, 3 µm.

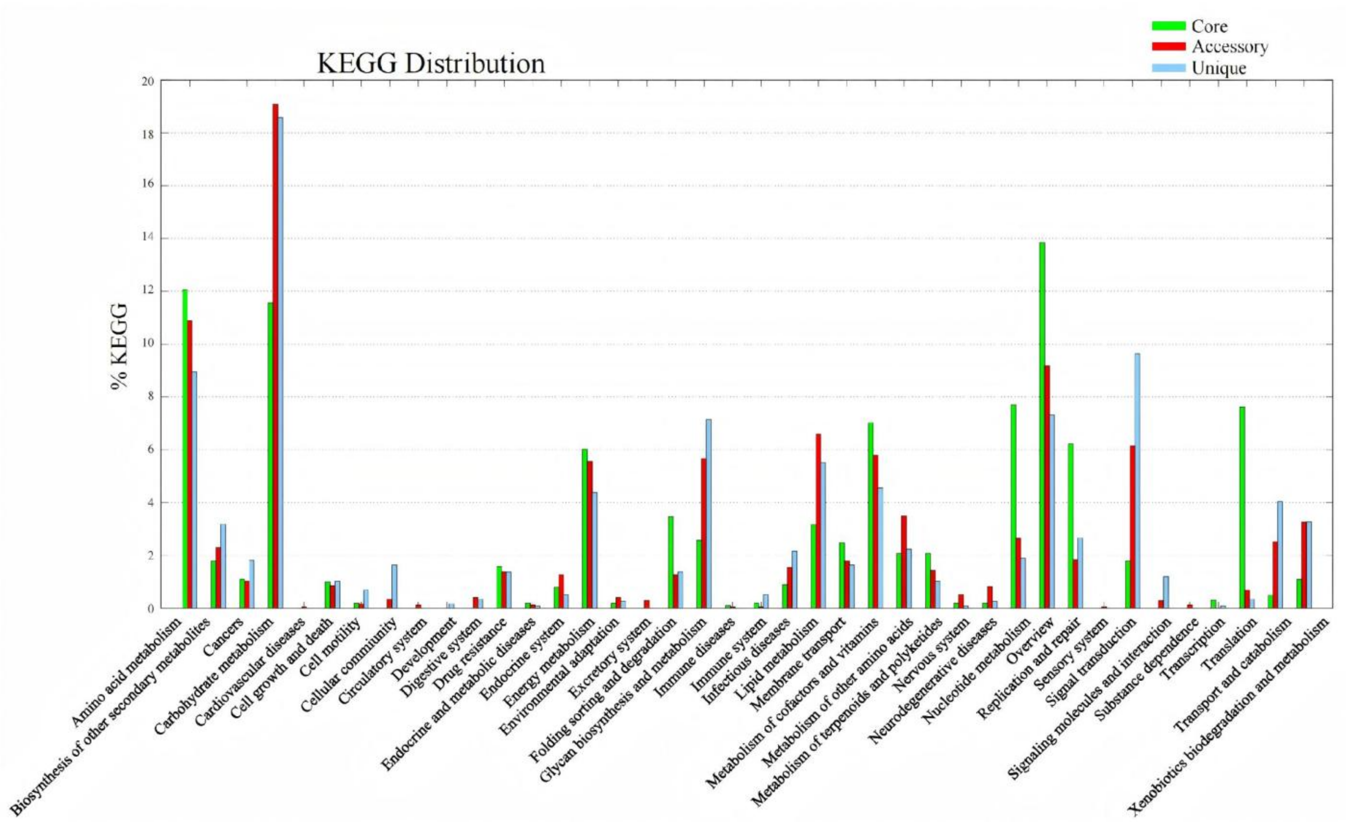

Figure S5. The distribution of core genes, accessory genes and unique genes to different metabolic pathways in the genus *Polaribacter*.

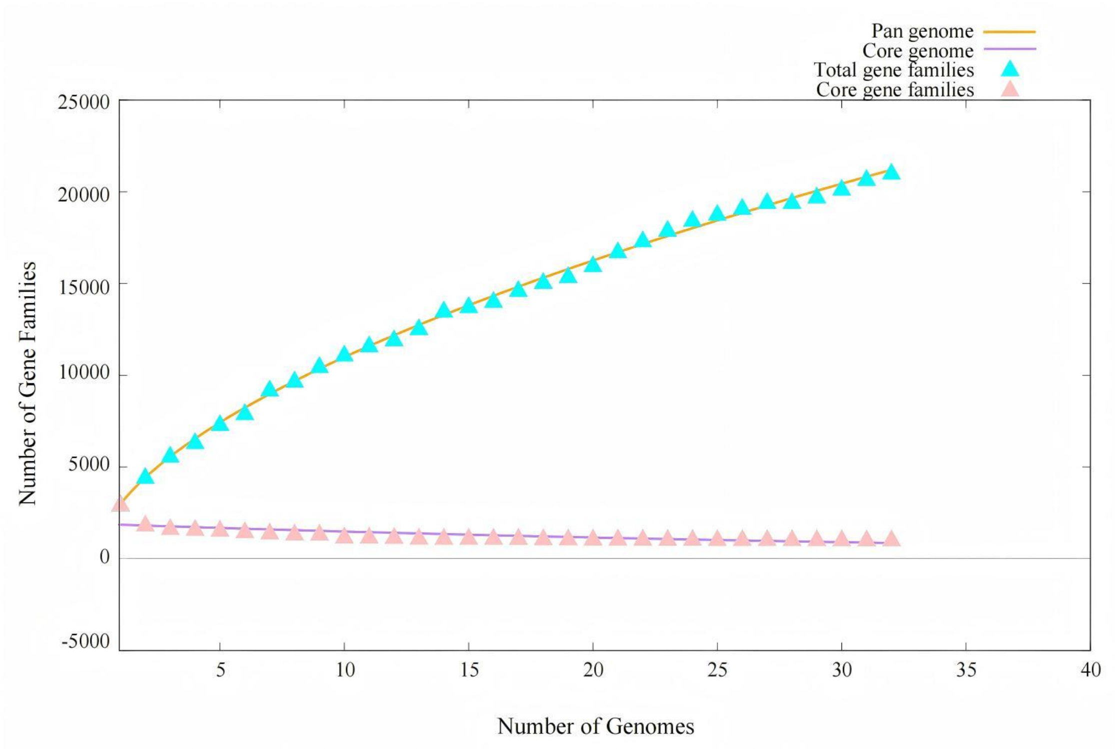

Figure S6. Pan-and core-genome of the genus *Polaribacter*. Triangle are the average of values obtained for the different strain combinations. Pan-genome power-fit curve equation: $f(x) = 2991.08x^{0.5650}$, core-genome exponential curve equation: $f1(x) = 1902.26e^{-0.0248x}$.

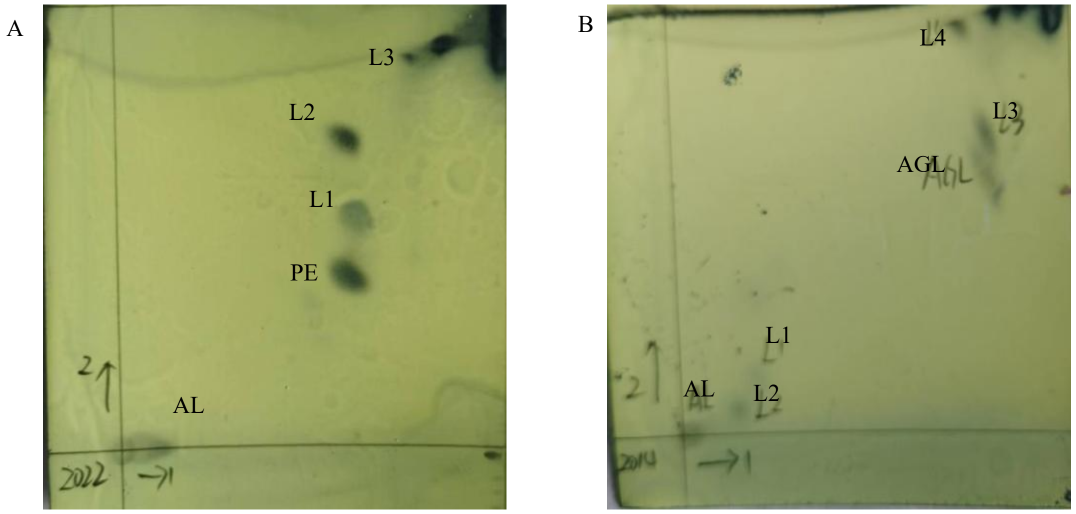

Figure S7. Two-dimensional TLC plate image of the total polar lipids of strain Z022^T (a) and Z014^T (b).

PE, phosphatidylethanolamine; AL, unidentified aminolipid; AGL, aminoglycolipid; L, unidentified lipid.

Table S2. The inhibition-zone diameters of strains Z022^T and Z014^T. The results are the mean of three replicates.

**antibiotics**	**antibiotic content(ug/piece)**	**Z014^T**	**Z022^T**
erythromycin	15	40	49
tobramycin	10	0	0
vancomycin	30	15	20
ampicillin	10	38	32
penicillin	10	40	37
chloramphenicol	30	33	45
Rifampin	5	56	37
tetracycline	30	10	21
norfloxacin	30	0	23
neomycin	30	0	0
Cefotaxime Sodium	30	40	33
gentamycin	10	0	0
ofloxacin	5	34	30
lincomycin	2	41	32
streptomycin	10	0	0
kanamycin	30	0	0
ceftriaxone	30	39	19
Polymyxin B	30	0	0
carbenicillin	100	34	50
clarithromycin	15	56	39

Table S3. List of phenotypic response to API 20E, API ZYM and API 50CHB identification systems (bioMérieux) and Biolog GEN III MicroPlates. Strains: 1, Z022^T; 2, Z014^T; 3, *Polaribacter filamentus* 215^T; 4, *Polaribacter atrinae* JCM 19202^T. All data are from this study. +, Positive; –, negative; w, weakly positive.

**Strain**		1	2	3	4
**API ZYM**					
2	alkaline phosphatase	+	+	+	+
3	esterase (C4)	+	+	–	w
4	esterase lipase (C8)	+	+	–	–
5	lipase (C14)	–	–	–	–
6	leucine arylamidase	+	+	+	+
7	valine arylamidase	+	+	+	+
8	cystine arylamidase	+	+	+	+
9	trypsin	+	+	+	+
10	*α*-chymotrypsin	+	+	–	–
11	acid phosphatase	+	+	+	+
12	naphthol-AS-BI-phosphohydrolase	+	+	+	+
13	*α*-galactosidase	–	–	–	+
14	*β*-galactosidase	–	–	–	–
15	*β*-glucuronidase	–	–	–	–
16	*α*-glucosidase	–	+	–	–
17	*β*-glucosidase	–	–	–	–
18	N-acetyl-*β*-glucosaminidase	+	–	–	+
19	*α*-mannosidase	–	–	–	–
20	*β*-fucosidase	+	–	–	–
**API 20E**					
ONPG	o-nitrophenyl-*β*-D-galactopyranoside	–	+	+	+

ADH	arginine dihydrolase	–	–	–	–
LDC	lysine decarboxylase	–	–	+	–
ODC	ornithine decarboxylase	–	–	–	–
CIT	citrate utilization	–	–	+	+
H2S	H_2S production	–	–	–	–
URE	Urease	–	–	–	–
TDA	tryptophan deaminase	–	–	–	+
IND	indole production	–	–	–	–
VP	Voges–Proskauer reaction	+	+	–	w
GEL	gelatinase	–	–	+	–
GLU	glucose	–	–	–	–
MAN	mannitol	–	–	–	–
INO	nositol	–	–	–	–
SOR	sorbitol	–	–	–	–
RHA	rhamnol	–	–	–	–
SAC	sucrose	–	–	–	–
MEL	melibiose	–	–	–	–
AMY	amygdalin	–	–	–	–
ARA	arabinose	–	–	–	–
**API 50CHB**					
1	glycerol	–	–	–	+
2	erythritol	–	–	–	–
3	D-arabinose	w	–	–	+
4	L-arabinose	–	–	–	+
5	D-ribose	+	–	–	+
6	D-xylose	w	+	–	+
7	L-xylose	w	–	–	–
8	D-adonitol	–	–	–	–
9	methyl-β-D-xylopyranoside	–	–	–	–

10	D-galactose	–	+	–	+
11	D-glucose	–	+	–	+
12	D-fructose	w	+	+	+
13	D-mannose	–	+	–	+
14	L-sorbose	+	–	–	–
15	L-rhamnose	–	–	–	–
16	dulcitol	–	–	+	–
17	inositol	–	–	+	–
18	D-mannitol	w	+	–	+
19	D-sorbitol	–	–	–	–
20	methyl-α-D-mannopyranoside	–	+	–	–
21	methyl-α-D-glucopyranoside	–	–	+	+
22	N-acetylglucosamine	–	–	–	+
23	amygdaline	–	+	–	+
24	arbulin	w	+	–	+
25	aesculin ferric citrate	w	+	+	+
26	salicin	–	–	–	–
27	D-cellobiose	–	+	–	+
28	D-maltose	–	+	–	+
29	D-lactose	–	+	–	+
30	D-melibiose	–	+	–	–
31	D-saccharose	–	–	–	+
32	D-trehalose	–	–	–	+
33	inulin	–	–	–	+
34	D-melezitose	–	+	–	+
35	D-raffinose	w	–	+	+
36	starch	–	+	–	+
37	glycogen	–	+	–	+
38	xylitol	–	–	+	–

39	gentiobiose	–	+	–	–
40	D-turanose	–	–	–	+
41	D-lyxose	w	–	–	w
42	D-tagatose	+	–	–	–
43	D-fucose	–	–	–	–
44	L-fucose	–	+	–	+
45	D-arabitol	–	–	–	–
46	L-arabitol	–	–	–	–
47	potassium gluconate	–	–	–	–
48	potassium 2-keto-gluconate	w	–	–	–
49	potassium 5-keto-gluconate	+	+	+	–
**Biolog GEN III MicroPlate**					
1A	negative-control	–	–	–	–
2A	dextrin	–	–	–	+
3A	D-maltose	–	–	–	+
4A	D-trehalose	–	–	–	+
5A	D-cellobiose	–	–	–	–
6A	gentiobiose	–	–	+	–
7A	sucrose	–	–	–	+
8A	D-turanose	–	–	–	–
9A	stachyose	–	–	–	–
1B	D-raffinose	–	–	–	+
2B	α-D-lactose	–	–	–	+
3B	D-melibiose	–	–	–	+
4B	β-methyl-D-glucoside	–	–	–	–
5B	D-salicin	–	+	–	–
6B	N-acetyl-D-glucosamine	–	–	–	+
7B	N-acetyl-β-D-mannosamine	–	–	–	+

8B	N-acetyl-D-galactosamine	–	–	–	–
9B	N-acetyl neuraminic acid	–	–	–	+
1C	α-D-glucose	–	+	–	+
2C	D-mannose	+	–	–	+
3C	D-fructose	–	–	–	+
4C	D-galactose	–	–	–	+
5C	3-methyl glucose	–	–	–	–
6C	D-fucose	–	–	–	–
7C	L-fucose	–	–	–	+
8C	L-rhamnose	–	–	–	–
9C	inosine	–	–	–	–
1D	D-sorbitol	–	–	–	–
2D	D-mannitol	–	–	–	+
3D	D-arabitol	–	–	–	+
4D	myo-inositol	–	+	–	–
5D	glycerol	–	–	–	–
6D	D-glucose-6-PO_4	–	–	–	+
7D	D-fructose-6-PO_4	–	+	–	–
8D	D-aspartic acid	–	–	–	–
9D	D-serine	–	–	–	–
1E	gelatin	–	–	–	+
2E	glycyl-L-proline	–	–	–	+
3E	L-alanine	–	–	–	–
4E	L-arginine	–	–	–	–
5E	L-aspartic acid	–	–	–	+
6E	L-glutamic acid	–	–	–	+
7E	L-histidine	–	–	–	–
8E	L-pyroglutamic acid	–	–	–	–
9E	L-serine	–	–	–	–

1F	pectin	–	–	–	–
2F	D-galacturonic acid	–	+	–	–
3F	L-galactonic acid lactone	–	+	–	–
4F	D-gluconic acid	–	–	–	–
5F	D-glucuronic acid	–	+	–	–
6F	glucuronamide	+	+	+	–
7F	mucic acid	–	–	–	–
8F	quinic acid	–	–	–	–
9F	D-saccharic acid	–	–	–	–
1G	ρ-hydroxy-phenylacectic acid	–	–	–	–
2G	methyl pyruvate	–	+	–	–
3G	D-lactic acid methyl ester	–	–	–	–
4G	L-lactic acid	–	+	–	–
5G	citric acid	–	–	–	–
6G	α-keto-glutaric acid	–	+	–	–
7G	D-malic acid	–	+	+	–
8G	L-malic acid	–	+	+	–
9G	bromo-succinic acid	–	–	–	–
1H	Tween-40	–	–	–	+
2H	γ-amino-butyric acid	–	–	–	–
3H	α-hydroxy-butyric acid	–	+	–	–
4H	β-hydroxy-D, L-butyric acid	+	+	–	–
5H	α-keto-butyric acid	–	–	–	–
6H	acetoacetic acid	–	+	–	+
7H	propionic acid	–	+	–	–
8H	acetic acid	–	–	–	–
9H	formic acid	+	–	–	+

Table S4. Cadmium resistance of strain Z022^T and Z014^T. Growth was assessed on marine agar supplemented with different concentrations of $CdCl_2$ (0–0.5 mM). Symbols: +++, good growth; ++, moderate growth; +, weak growth; −, no growth.

Concentration of $CdCl_2$ (mM)	Z022^T	Z014^T
0	+++	+++
0.1	–	+++
0.2	–	++
0.3	–	+
0.4	–	–
0.5	–	–

Table S6. Cellular fatty acid composition (%) of strain Z022[T], Z014[T] and the closely related type strains.

**Fatty acid**	1	2	3	4	5[a]
**Saturated**					
$C_{13:0}$	0.51	0.66	0.72	2.32	TR
$C_{14:0}$	2.40	1.44	TR	1.18	TR
$C_{16:0}$	2.04	TR	0.65	0.64	–
**Unsaturated**					
$C_{15:1}\omega6c$	3.90	8.87	4.67	**17.22**	**12.2**
$C_{16:1}\omega5c$	0.76	0.76	TR	0.87	–
$C_{17:1}\omega6c$	–	0.52	0.64	2.57	TR
**Branched**					
anteiso-$C_{13:0}$	0.61	0.82	TR	0.77	–
anteiso-$C_{15:0}$	1.29	9.61	2.62	5.36	1.9
anteiso-$C_{15:1}$ A	–	2.32	–	1.18	–
iso-$C_{13:0}$	9.89	8.90	4.55	9.09	3.2
iso-$C_{14:0}$	–	2.30	2.06	2.07	6.5
iso-$C_{15:0}$	**43.90**	**11.73**	**18.94**	**12.15**	**19.7**
iso-$C_{15:1}$ G	–	9.27	**11.97**	8.80	9.5
**Hydroxy**					
iso-$C_{15:0}$3-OH	**16.80**	**18.49**	**31.73**	**13.17**	**12.4**
$C_{15:0}$ 2-OH	TR	1.88	0.86	0.89	TR
$C_{15:0}$ 3-OH	–	**10.36**	4.70	8.33	4.8
iso-$C_{16:0}$3-OH	–	1.71	3.07	1.16	5.5
$C_{16:0}$ 3-OH	–	2.05	0.71	2.04	TR
**Summed features**					
3	3.43	3.12	TR	5.05	**12.5**

Strains: 1, Z022^T; 2, Z014^T; 3, *Polaribacter filamentus* KCTC 23135^T; 4, *Polaribacter atrinae* JCM 19202^T; 5, *Polaribacter ponticola* KACC 22340^T. Data represent the percent of the total fatty acids as determined by the Microbial Identification System software. All data were obtained from this study. Fatty acids that represented <0.5% in all columns were omitted. Fatty acids that represented >10.0% are indicated in bold type. TR, Traces (<0.5%); –, not detected.

Summed features are groups of two or three fatty acids that cannot be separated by GLC using the MIDI system. Summed feature 3 consists of $C_{16:1}$ ω7c and/or $C_{16:1}$ ω6c.

a: Data from Baek *et al.* (2024).
